# Jasmonate-dependent plant defense restricts thrips performance and preference

**DOI:** 10.1186/1471-2229-9-97

**Published:** 2009-07-27

**Authors:** Hiroshi Abe, Takeshi Shimoda, Jun Ohnishi, Soichi Kugimiya, Mari Narusaka, Shigemi Seo, Yoshihiro Narusaka, Shinya Tsuda, Masatomo Kobayashi

**Affiliations:** 1 Experimental Plant Division, RIKEN BioResource Center, Tsukuba 305-0074, Japan; 2 National Agricultural Research Center, Tsukuba 305-8666, Japan; 3 National Institute of Vegetable and Tea Science, Tsu 514-2392, Japan; 4 National Institute for Agro-Environmental Sciences, Tsukuba 305-8604, Japan; 5 Research Institute for Biological Sciences, Okayama 716-1241, Japan; 6 National Institute of Agrobiological Sciences, Tsukuba 305-8666, Japan

## Abstract

**Background:**

The western flower thrips (*Frankliniella occidentalis *[Pergande]) is one of the most important insect herbivores of cultivated plants. However, no pesticide provides complete control of this species, and insecticide resistance has emerged around the world. We previously reported the important role of jasmonate (JA) in the plant's immediate response to thrips feeding by using an *Arabidopsis *leaf disc system. In this study, as the first step toward practical use of JA in thrips control, we analyzed the effect of JA-regulated *Arabidopsis *defense at the whole plant level on thrips behavior and life cycle at the population level over an extended period. We also studied the effectiveness of JA-regulated plant defense on thrips damage in Chinese cabbage (*Brassica rapa *subsp. *pekinensis*).

**Results:**

Thrips oviposited more on *Arabidopsis *JA-insensitive *coi1-1 *mutants than on WT plants, and the population density of the following thrips generation increased on *coi1-1 *mutants. Moreover, thrips preferred *coi1-1 *mutants more than WT plants. Application of JA to WT plants before thrips attack decreased the thrips population. To analyze these important functions of JA in a brassica crop plant, we analyzed the expression of marker genes for JA response in *B. rapa*. Thrips feeding induced expression of these marker genes and significantly increased the JA content in *B. rapa*. Application of JA to *B. rapa *enhanced plant resistance to thrips, restricted oviposition, and reduced the population density of the following generation.

**Conclusion:**

Our results indicate that the JA-regulated plant defense restricts thrips performance and preference, and plays an important role in the resistance of *Arabidopsis *and *B. rapa *to thrips damage.

## Background

Insect attack is one of the most important factors retarding plant growth, decreasing crop productivity, and causing other agricultural problems. A constitutive and inducible plant defense response confers immunity to herbivorous insects [1-3]. Analyses at the molecular, metabolic, and physiological levels [2,4] have focused on responses to lepidopteran larvae (caterpillars) and aphids. Many analyses of plant responses to feeding by caterpillars have been conducted [e.g., [5-7]]. Caterpillars harm plants by chewing-type feeding, the best understood of several feeding modes. Although caterpillar feeding and mechanical wounding are physically similar, plants show obvious specific responses to caterpillar feeding [8]. Some of these responses are induced by insect gut and oviposition [9,10]. The sucking-type feeding by aphids and whiteflies is also well understood. However, in contrast to caterpillar feeding, sucking-type feeding rarely causes mechanical damage to the host plant. Rossi et al. [11] reported that the nematode resistance (*R*) gene *Mi-1 *of tomato is involved in resistance to the potato aphid. *Mi-1 *also confers resistance to whiteflies [12]. Other major classes of insect feeding are also known. Leafminers feed within leaves and stems, forming tunnels (mining-type feeding), and thrips and spider mites feed by piercing and sucking [13,14].

The western flower thrips (*Frankliniella occidentalis *[Pergande]) is one of the most important insect herbivores. This tiny insect tends to occupy narrow crevices within or between plant parts. The emergence worldwide of insecticide resistance among western flower thrips makes them difficult to control [15]. The thrips can also act as a vector of tospoviruses such as tomato spotted wilt virus [16,17]. Damage by western flower thrips is increasing in many countries; in particular, injury in greenhouse production is serious [18-20]. Thus, the development of new methods to control thrips damage by using the molecular mechanisms of plant responses is needed.

Jasmonate (JA) has an important function in plant responses to caterpillars and aphids [2]. Reymond et al. [21] reported that the JA-insensitive *coi1-1 *mutant of *Arabidopsis *is less resistant to cabbage butterfly (*Pieris rapae*). Ellis et al. [22] reported that *coi1-1 *mutants are less resistant to aphids, but the constitutive JA-signaling mutant *cev1 *is more resistant. Our recent study focusing on *Arabidopsis *response to thrips feeding also indicated the important function of JA [23,24], and comparative transcriptome analyses suggested a strong relationship between JA treatment and thrips feeding [23]. Several groups reported that JA-regulated gene expression is induced by spider mites feeding [25,26], which have a similar feeding mode to that of thrips. De Vos et al., using *Arabidopsis *genome arrays [27], also reported the importance of JA for feeding-inducible gene expression by thrips and cabbage butterfly attack. Interestingly, they indicated the existence of common genes in the response to both feeding modes, and genes specific to each feeding mode.

*Arabidopsis *is a widely studied experimental plant for which many useful genomic resources and much other information are available. However, it is not suitable for analyzing *Arabidopsis *responses to caterpillars, which can quickly eat an entire plant. On the other hand, with the tiny western flower thrips, it is possible to analyze *Arabidopsis *responses to thrips attack over generations.

In this study, we focused on the effect of JA-regulated *Arabidopsis *defense at the whole plant level on thrips behavior and life cycle at the population level. We analyzed the long-term effects of JA-regulated plant defense on thrips oviposition, the population density of the following thrips generation (larvae and pupae), and preference between *Arabidopsis *WT and JA-insensitive *coi1-1 *mutant host plants. The results show important effects of the JA-dependent plant defense on both thrips performance and preference. In addition, application of JA to *Arabidopsis *WT plants before thrips attack decreased the thrips population. Expression analyses of marker genes for JA response in Chinese cabbage (*Brassica rapa *subsp. *pekinensis*) suggested the occurrence of a JA-dependent defense against thrips attack in this plant, too. The JA content of *B. rapa *was significantly increased after thrips feeding, and application of JA to plants enhanced their resistance to thrips.

## Results

### Importance of jasmonate-regulated *Arabidopsis *defense in resistance to thrips attack

**Figure 1:**
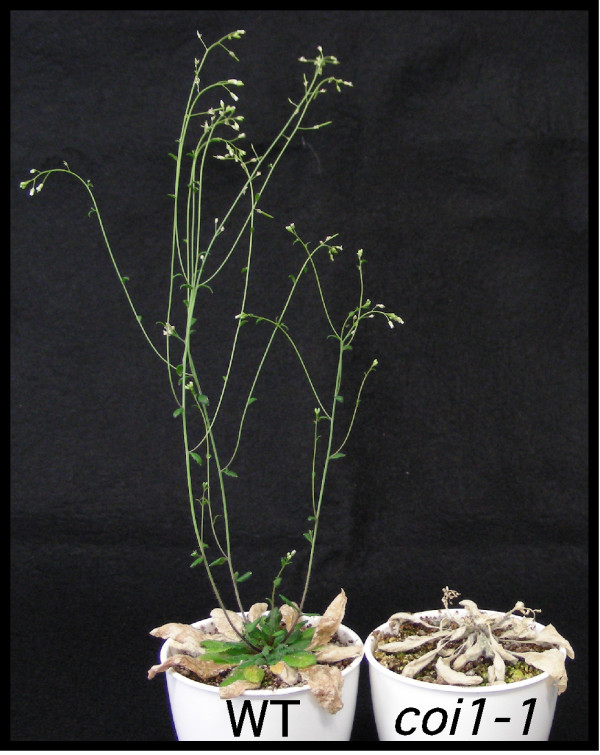
**Function of JA in plant resistance to thrips feeding**. Twenty adult females fed on 3-week-old WT plants (left) or *coi1-1 *mutants (right). Typical plants after 4 weeks of feeding are shown.

We recently reported the role of JA in the short-term response of *Arabidopsis *to thrips feeding on leaf discs over 1 or 2 days [23,24]. To analyze its role in long-term defense at the whole plant level, we compared the feeding damage between whole WT plants and JA-insensitive *coi1-1 *mutants [28] inoculated with 20 thrips at 3 weeks. The *coi1-1 *mutants had been completely devoured by 4 weeks after inoculation, whereas WT plants were flowering and producing siliques (Fig. 1A). These results suggest the importance of JA-regulated defense in the resistance of *Arabidopsis *to thrips attack.

**Figure 2:**
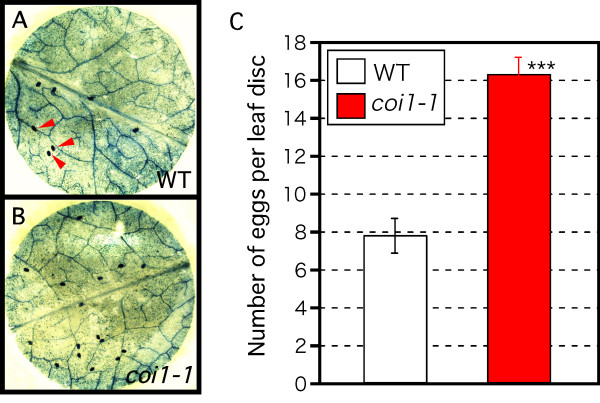
**Effect of JA-dependent plant resistance on thrips oviposition on leaf discs**. One adult female fed per leaf disc of 3-week-old WT plants (A) or *coi1-1 *mutants (B) for 4 days. Eggs oviposited on leaf discs were stained with trypan blue. Photos show typical leaf discs after staining; some eggs are shown by red arrowheads. (C) Number of eggs per leaf disc (mean ± SD) based on more than 10 independent determinations. Asterisks indicate significant difference (Student's *t*-test), ***p < 0.001.

To understand why *coi1-1 *mutants showed low resistance to thrips attack, we first analyzed the number of thrips eggs on the WT plants and *coi1-1 *mutants to compare the asexual oviposition performance of thrips. *Arabidopsis *rosette leaves were cut into leaf discs with 8-mm diameter. One adult female thrips was put on each disc and allowed to feed and oviposit. Because the females lay in the epidermal or mesophyll cells [29], we stained the eggs with trypan blue to count them. As we reported previously [23], the area of feeding scars on *coi1-1 *mutants was greater than that on WT plants (data not shown). The number of eggs on the *coi1-1 *discs was double that on the WT discs (Fig. 2A–C). The decreased resistance of these *coi1-1 *mutants could explain the increased oviposition rate on these plants.

### Effect of jasmonate-dependent *Arabidopsis *defense on thrips population

**Figure 3:**
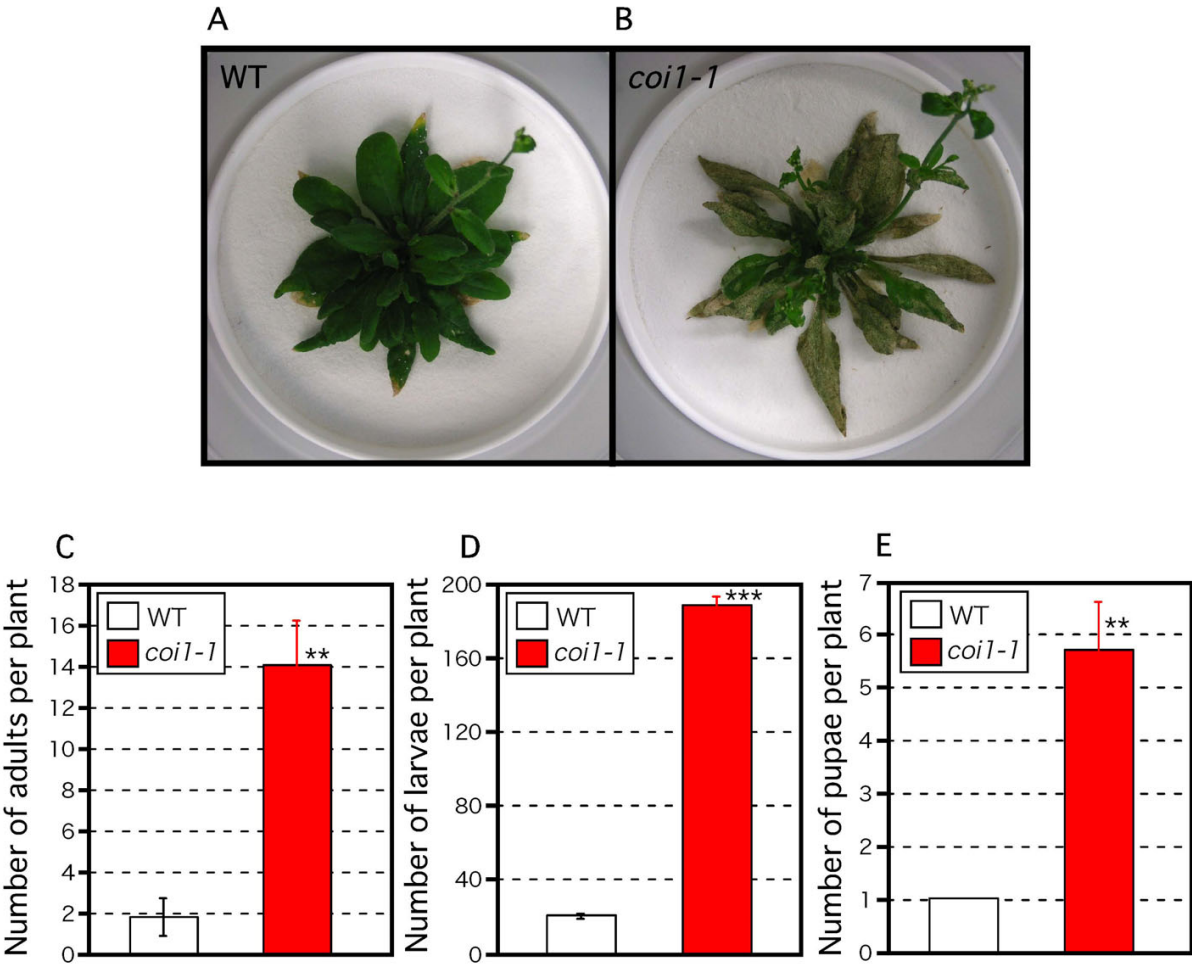
**Effect of the JA-dependent plant defense on thrips population**. (A, B) Twenty adult females fed on 3-week-old WT plants (A) or *coi1-1 *mutants (B) for 2 weeks. (C-E) Number of adults (C), larvae (D), and pupae (E) after 2 weeks; mean ± SD based on five independent determinations. Asterisks indicate significant differences (Student's *t*-test), **p < 0.005, ***p < 0.001.

Because the JA-regulated defense affected oviposition, we analyzed its effect on the subsequent generation. We put 20 adult females on WT and *coi1-1 *plants and counted adults, larvae, and pupae after 2 weeks. We covered the soil with fine zirconia beads 0.4 mm in diameter to find thrips easily. Thrips fed much more on *coi1-1 *mutants than on WT plants (Fig. 3A, B). About 14 of the original adult females remained on *coi1-1 *mutants, but only about 2 remained on WT plants (Fig. 3C). Similarly, while more than 190 larvae lived on the *coi1-1 *mutants, only about 20 lived on the WT plants (Fig. 3D). We also found 5 times as many pupae on *coi1-1 *mutants than on WT plants (Fig. 3E). These results demonstrate that the JA-regulated defense can determine thrips population size.

**Figure 4:**
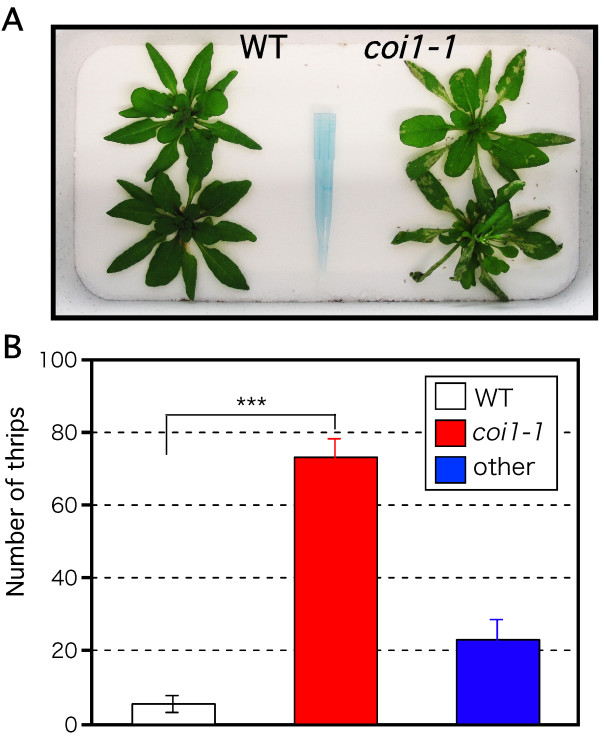
**Effect of the JA-dependent plant defense on host plant preference of thrips**. (A) Three-week-old WT plants (left) and *coi1-1 *mutants (right) were grown at each end of a pot. One hundred adult females were collected in a 1-mL tube and laid between the plants. Photo shows plants after 2 days. (B) Number of adult thrips on each plant after 2 days. Mean ± SD based on five independent determinations. Asterisk indicates a significant difference between the two plants, *χ*^2 ^test, ***p < 0.001. The remaining thrips roamed the surroundings were excluded from the statistical analysis.

Next, we analyzed the effect of JA-regulated plant defense on host plant preference of thrips. We placed 100 adult females halfway between WT and *coi1-1 *plants (Fig. 4A) and counted the thrips on each plant after 2 days. The *coi1-1 *mutants had many more thrips than the WT plants (Fig. 4A): > 70% versus about 5% (*χ*^2 ^test, *χ*^2 ^= 175.879, df = 1, p < 0.001; Fig. 4B); the remaining thrips roamed the surroundings. These results indicate that the JA-regulated plant defense influences the host plant preference of thrips.

**Figure 5:**
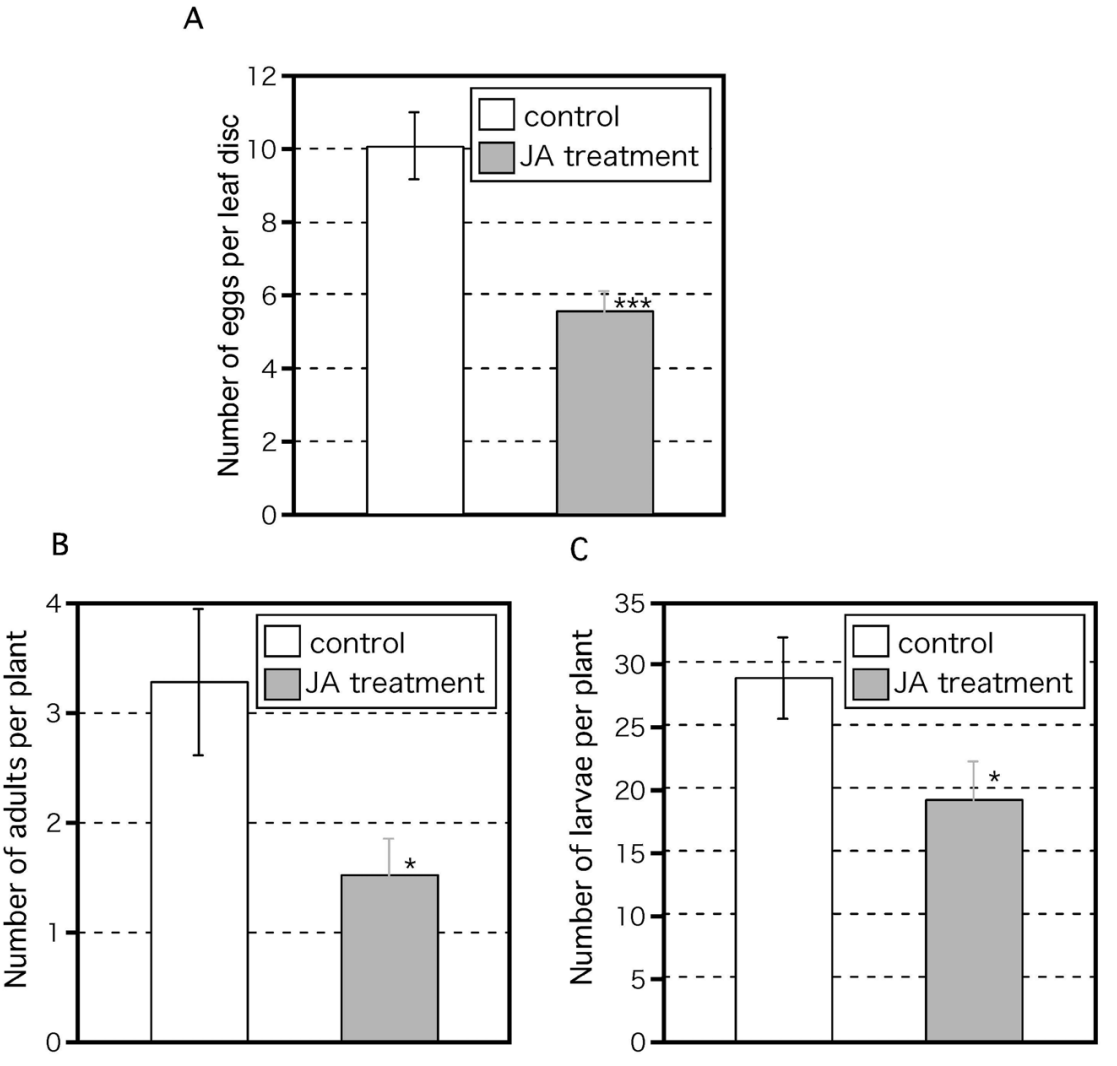
**Effect of JA-induced *Arabidopsis *defense response on thrips population**. Twenty adult females fed on 3-week-old WT plants. Either 50 μM JA or water (control) was applied 2 days before thrips were introduced. After 2 weeks, eggs (A), adults (B), and larvae (C) were counted. Mean ± SD based on five independent determinations. Asterisks indicate significant differences (Student's *t*-test), *p < 0.05, ***p < 0.001.

We next analyzed the effect of JA treatment on *Arabidopsis *resistance to thrips attack. JA-treated plants had half as many eggs as untreated plants (Fig. 5A). The numbers of adults and larvae showed a similar contrast (Fig. 5B, C). Together with the results from the *coi1-1 *mutants, these results indicate that the JA-dependent defense response in *Arabidopsis *plays an important role in resisting thrips.

### Jasmonate-dependent plant resistance to thrips in *B. rapa*

**Figure 6:**
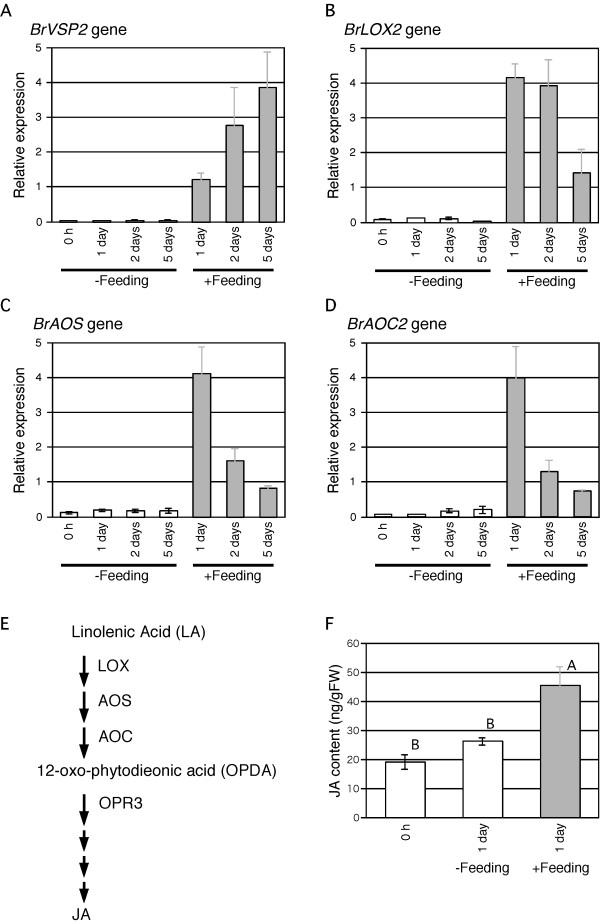
**Involvement of JA signaling in *B. rapa *response to thrips feeding**. (A-D) Expression of marker genes for JA response in *B. rapa *was induced by thrips feeding. *BrVSP2 *(A), *BrLOX2 *(B), *BrAOS *(C), and *BrAOC2 *(D) are brassica counterparts of *Arabidopsis *marker genes of the JA pathway and JA biosynthesis. Twenty-five adult females fed on five 2-week-old plants per pot. After 0, 1, 2, and 5 days, total RNA was prepared from the plants with (+Feeding) or without (-Feeding) thrips, and first-strand cDNA was synthesized for PCR analysis. The expression level of each gene was normalized to the expression of *BrACT2 *(control). Mean ± SD based on three replications. (E) Proposed model of the biosynthesis of JA in *Arabidopsis*. (F) Effect of thrips feeding on the biosynthesis of JA in *B. rapa*. Ten adult females fed on a 2-week-old plant (+Feeding). A control plant was kept without thrips (-Feeding). At the beginning of the experiment (0 h) and after 1 day from the start of feeding, 1 g of plant tissue was sampled for measurement of endogenous JA (JA + methyl JA). Means ± SD of three independent measurements. Different letters indicate statistically significant differences between treatments (Tukey-Kramer HSD test; p < 0.05).

To search for JA-dependent resistance to thrips in a brassica crop, we analyzed the function of JA in *B. rapa*, one of the most important brassica crops in the world, especially in Asia. A search of the *B. rapa *EST database (National Center for Biotechnology Information) revealed putative counterparts of *Arabidopsis *JA-inducible marker genes. We analyzed the expression of marker genes of the JA pathway corresponding to *AtVSP2 *and *AtLOX2*, and genes corresponding to *allene oxide synthase *(*AtAOS*) and *allene oxide cyclase 2 *(*AtAOC2*), both of which encode enzymes that catalyze JA biosynthesis as shown by previous reports (Fig. 6E) [30,31]. Expression of the brassica counterparts, *BrVSP2*, *BrLOX2*, *BrAOS*, and *BrAOC2*, was induced by thrips feeding (Fig. 6A–D). In addition, the JA content of *B. rapa *plants infested by thrips was significantly higher than that of control plants (one-way ANOVA, *F *= 13.938, df = 2, p < 0.01; Fig. 6F). These data suggest the involvement of JA in the response to thrips feeding in *B. rapa *also.

**Figure 7:**
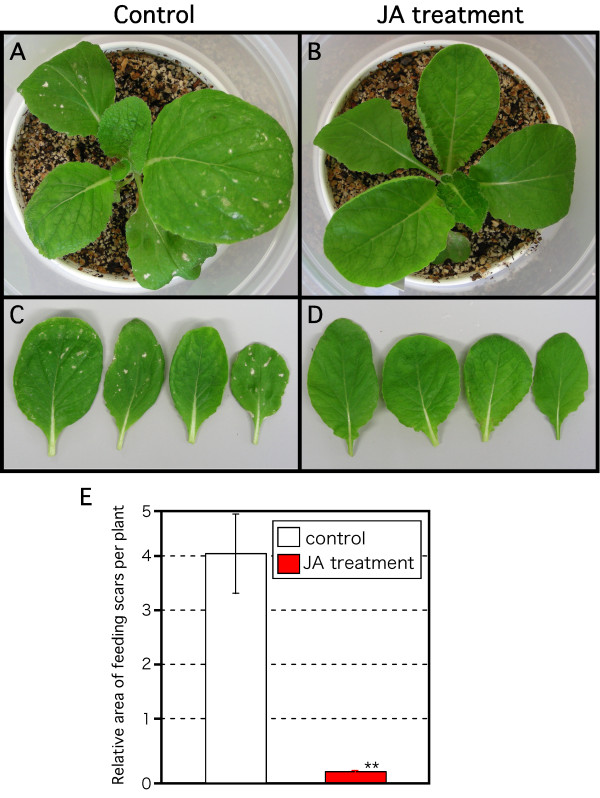
**Effect of JA application on plant resistance to thrips**. Twenty adult females fed on 2-week-old *B. rapa *plants for 10 days. Water (control; A, C) or 50 μM JA (B, D) was applied 1 day before thrips were introduced. (E) Mean ± SD of area of feeding scars based on more than 10 independent determinations. Asterisk indicates significant difference (Student's *t*-test), **p < 0.005.

To confirm the functional role of JA in plant resistance to thrips attack in *B. rapa*, we analyzed the effect of JA treatment on thrips feeding. Injury from thrips attack was lower in plants treated with JA than in untreated plants (Fig. 7A–D), by a factor of about 15 (Fig. 7E). These results indicate that the JA-dependent plant defense against thrips is conserved in *B. rapa*.

**Figure 8:**
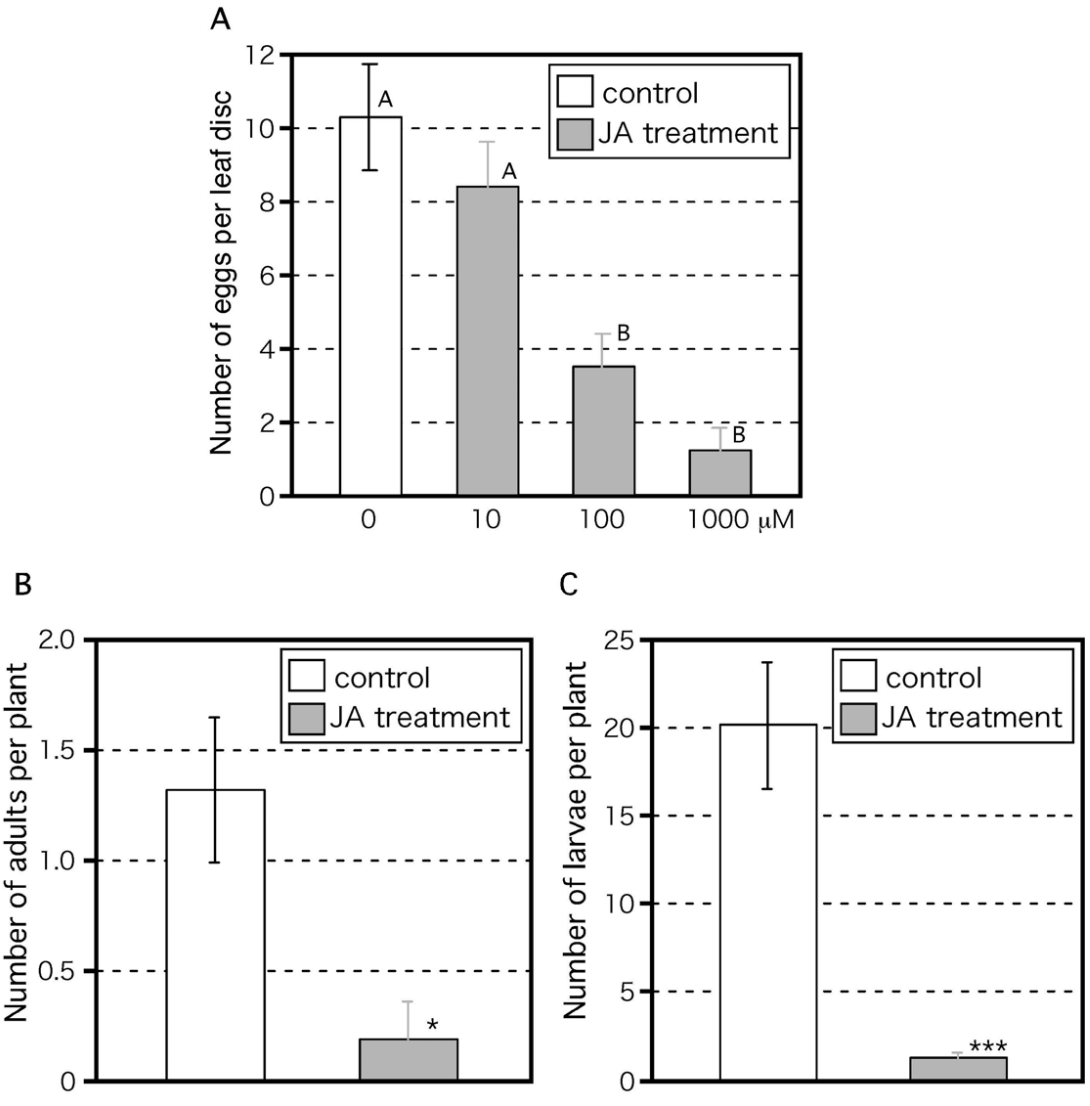
**Effect of JA-induced *B. rapa *defense response on thrips population**. (A) Water (control) or 10, 100, or 1000 μM JA was applied to 2-week-old *B. rapa *plants 1 day before thrips were introduced. One adult female fed on each leaf disc for 4 days. Eggs were stained with trypan blue. Mean ± SD of eggs per leaf disc based on 10 independent determinations. Different letters indicate statistically significant differences between treatments (Tukey-Kramer HSD test; p < 0.05). (B, C) Twenty adult females fed on 2-week-old WT plants for 2 weeks. Water (control) or 50 μM JA was applied 1 day before thrips were introduced. After 2 weeks, adults (B) and larvae (C) were counted. Mean ± SD based on five independent determinations. Asterisks indicate significant differences (Student's *t*-test), *p < 0.05, ***p < 0.001.

We further analyzed JA's effect on thrips oviposition. Rosette leaves of *B. rapa *were cut into leaf discs with 8-mm diameter. One adult female thrips was put on each leaf disc and allowed to feed and oviposit for 4 days. Application of JA dose-dependently decreased the number of eggs (one-way ANOVA, *F *= 10.367, df = 4, p < 0.001; Fig. 8A). Finally, we analyzed the effect of JA on the next generation. JA treatment of plants restrained the thrips population very effectively (Fig. 8B, C). These results clearly indicate the important role of JA in resistance to thrips attack in *B. rapa *also.

## Discussion

The phytohormone JA regulates part of a plant's basal defense system. Numerous studies have examined the functions of JA in plant responses to pathogen attack, mechanical wounding, UV irradiation, ozone exposure, osmotic stress [32,33], and insect feeding [34,35]. The JAZ (jasmonate ZIM-domain) family of repressors was identified in *Arabidopsis *as a negative regulator of JA signaling [36-38]. JAZ interacts with COI1 protein, degrades, and so induces JA-responsive gene expression. Overexpression of a modified form of JAZ1 significantly decreased plant resistance to the beet armyworm (*Spodoptera exigua*) [39]. Resistance by *coi1-1 *mutants to cabbage butterfly caterpillar (*Pieris rapae*) was similarly decreased [21].

However, these analyses focused on plant responses to lepidopteran larvae. Because caterpillars quickly devour *Arabidopsis *plants and change to butterflies or moths, which fly away, it is difficult to analyze the *Arabidopsis *response and insect performance over generations on the one *Arabidopsis *plant. For these reasons, we used thrips. We found differences in symptoms between WT plants and JA-insensitive *coi1-1 *mutants: thrips had demolished *coi1-1 *mutants after 4 weeks, yet WT plants had flowers and siliques (Fig. 1A). As it seemed unlikely that only 20 adult thrips could kill a plant in 4 weeks, we also studied the effect of a JA-dependent *Arabidopsis *defense on oviposition. The number of eggs on *coi1-1 *was about double that on the WT (Fig. 2A–C). As we described previously [23], the area of feeding scars in *coi1-1 *was much greater than that on WT plants (data not shown). The greater number of eggs on *coi1-1 *might result from the better performance of adult thrips. Alternatively, a difference in plant metabolites between WT and *coi1-1 *might influence oviposition. Annadana et al. [40] reported that cysteine protease inhibitors restrict oviposition by western flower thrips. Wounding and JA induce many genes encoding cysteine protease inhibitors [41], including *Arabidopsis *cystatin-1 (AtCYS1) [42]. Cysteine protease inhibitors could explain the difference in thrips oviposition between WT and *coi1-1 *plants.

Next, we analyzed the effect of JA-regulated plant defense on the population density of the following generation of thrips. Surprisingly, the population increased around 10-fold after 2 weeks on the *coi1-1 *mutants, but changed little on the WT plants (Fig. 3A–E). Most of the thrips on *coi1-1 *were larvae. We found some dead larvae on the WT plants but none on *coi1-1 *(data not shown). These results indicate that the JA-dependent plant defense in WT plants reduces the survival of thrips larvae. We found about 7 times as many adult thrips on *coi1-1 *as on the WT, which indicates that thrips can survive longer on *coi1-1*. We attribute the much greater population of thrips on *coi1-1 *to this increased longevity and the greater egg production on *coi1-1 *mutants, and the higher mortality of larvae on the WT plants. Analysis of the hatching rate of eggs could also help explain the increased population on *coi1-1*. Barth et al. [43] reported that a double knock-out mutant of *Arabidopsis *lacking two major genes for myrosinase (*tgg1*, *tgg2*), which degrades glucosinolates to toxins such as isothiocyanates, showed decreased resistance to the cabbage looper (*Trichoplusia ni*) and tobacco hornworm (*Manduca sexta*). Sasaki-Sekimoto et al. [33] reported that JA regulates glucosinolate biosynthesis. Recently, Shroff et al. [44] showed that the preferential allocation of glucosinolates to the periphery of leaves may play a key role in the defense of leaves by creating a barrier to chewing herbivores, which frequently approach leaves from the edge. Several other compounds protect plants against insect pests. Konno et al. [45] reported that cysteine proteases such as papain, ficin, and bromelain showed toxicity to two notorious pests, cabbage armyworm (*Mamestra brassicae*) and cotton leafworm (*Spodoptera litura*). They later reported that sugar-mimic alkaloids were toxic to cabbage armyworm [46]. Further analyses will help to explain which kinds of compounds, regulated by JA, reduce thrips performance.

The choice test showed that *coi1-1 *mutants attracted 14 times as many thrips as did WT plants (Fig. 4A, B). As a result, *coi1-1 *mutants suffered more damage. Aharoni et al. [47] reported that overexpression of a gene for a dual linalool/nerolidol synthase (*FaNES1*) in *Arabidopsis*, which produces those two terpenes, enhances avoidance by green peach aphids (*Myzus persicae*). Interestingly, these *FaNES1*-overexpressing plants also attracted carnivorous predatory mites (*Phytoseiulus persimilis*) [48]. JA-deficient *spr2 *tomato plants emit less herbivory-induced volatiles and attract more tobacco hornworm and tobacco whitefly (*Bemisia tabaci*) for oviposition [49]. In addition to the volatile components, many other plant metabolites such as nutrient factors and toxic compounds are reported as stimulants or deterrents of host plant preference [50]. These metabolic components may explain the higher preference of the thrips for *coi1-1 *mutants or higher avoidance of WT plants.

The western flower thrips is one of the most serious insect herbivores in the world. It is also a virus vector. Because of its thigmokinetic behavior and the emergence of insecticide resistance, it is difficult to control with insecticides [15]. Therefore, new control methods are urgently needed. Application of JA to WT *Arabidopsis *plants before thrips damage decreased the thrips population (Fig. 5A–C). We previously reported that thrips feeding induced in *Arabidopsis *expression of *AtVSP2 *and *AtLOX2 *(marker genes of the JA pathway) and *AtAOS1 *and *AtAOC2 *(encoding allene oxide synthase and allene oxide cyclase), which catalyze JA biosynthesis in *Arabidopsis *[23]. Here, the expression of their counterparts in *B. rapa *was also induced by thrips feeding (Fig. 6A–D), as was the JA content (Fig. 6F), as reported previously in *Arabidopsis *[23]. These results indicate that the JA-dependent defense system is conserved between *Arabidopsis *and *B. rapa*. Interestingly, JA application also greatly decreased the amount of feeding scars in *B. rapa *(Fig. 7A–E), and decreased egg production and thrips population size (Fig. 8A–C). The effect of JA application was much higher in *B. rapa *than in *Arabidopsis*, but the biological significance of this difference is unclear. Several groups have combined JA-mediated transcriptome analyses with metabolomics data [33,51]. Further comparative analyses between *B. rapa *and *Arabidopsis *using these approaches are needed to explain the differences in plant resistance. The genome of *B. rapa *is being sequenced . In the near future, *Brassica *'omics' analyses using genome information will be available. Comparative expression analyses between *B. rapa *and *Arabidopsis *suggested the existence of similar and specific responses to pathogen infection in these species [52].

Jasmonate application to *Nicotiana sylvestris *plants decreased plant biomass [53]. Overexpression of *AtJMT *in *Arabidopsis *plants, which leads to elevated JA level [54], decreased the flower number and total seed weight significantly. Importantly, Thaler et al. showed that although application of JA in tomato fields successively decreased naturally occurring thrips, spray application at low concentration (0.5 mM) decreased neither plant biomass nor fruit production [55]. However, the effect of low JA concentration on thrips control is lower than that of high JA concentration (1.5 mM). JA application incurs costs for plant fitness, and also activates plant defense, which must be balanced for optimum production. The screening of the specific compounds to regulate plant defense to insect attack will be a promising approach.

## Conclusion

In this study, as the first step toward practical use of JA in thrips control, we analyzed the effect of JA-regulated *Arabidopsis *defense at the whole plant level on thrips behavior and life cycle at the population level. Our results indicate that JA-regulated *Arabidopsis *defense restricts both thrips performance and preference. Thrips performance was evaluated from oviposition and the population density of the following generation. The effect of JA-regulated defense on thrips population density was considerable. This was due to the effects on thrips longevity, egg production, and mortality of larvae. Fully understanding the plant defense against thrips attack will require determination of the actual plant metabolites that restrict thrips performance and preference.

In *B. rapa *also, induction of expression of marker genes for the JA pathway and increased JA content after thrips damage support the occurrence of a JA-dependent defense against thrips attack. JA application to *B. rapa *greatly decreased feeding damage on account of decreased egg production and thrips population density. The existence of diverse targets of JA-regulated plant defense indicates that JA concurrently regulates multiple responses involved in plant resistance to thrips damage. JA-regulated plant defense could be a good target for practical applications to control thrips.

## Methods

### Plant materials and cultivation

Wild-type (ecotype Col-0) *Arabidopsis *plants and the JA signaling and biosynthesis mutant *coi1-1 *were grown in soil as described previously [56]. Briefly, seeds were sown on sterile soil in pots, moistened, and held at 4°C for 7 days in the dark to synchronize germination. The pots were then transferred to 22°C with a long-day photoperiod (16 h light/8 h dark). Plants at the four-leaf stage were transferred individually to pots and grown to the rosette stage. Chinese cabbage (*B. rapa *subsp. *pekinensis *cv. Kyoto No. 3, Takii Seed Co. Ltd., Kyoto, Japan) plants were grown similarly.

### Identification of *coi1-1 *plants

Homozygous *coi1-1 *plants were selected according to PCR amplification of a sequence of the *Arabidopsis COI1 *gene followed by digestion with *Bsm*I (TOYOBO, Osaka, Japan). Within the amplified PCR product, the *Bsm*I restriction site is present only in the *coi1-1 *mutant. Primers were as follows: forward, 5'-GGAAACAGGAGCCCGAGATC-3'; reverse, 5'-TGGATGTTTCTCGGAGCAGC-3'.

### Thrips attack

Laboratory colonies of *Frankliniella occidentalis *were maintained in a closed environmental chamber, as described previously [57]. The assay used female adults 14–21 days after emergence from the pupal stage. The adults were starved for 2 to 3 h before feeding on test plants. Twenty adult females were allowed to feed on each whole plant in a cylindrical acryl chamber with air ventilation windows covered with a fine mesh.

### Jasmonate treatment

Pots holding 3-week-old *Arabidopsis *plants or 2-week-old *B. rapa *plants grown in soil were transferred into a cylindrical acryl chamber containing 100 μM JA solution. Other experiments to count the number of eggs on *B. rapa *leaf discs used 10, 100, or 1000 μM JA solution. JA treatment was carried out for 2 days before the beginning of thrips attack.

### Counting of thrips eggs

Leaf discs with 8-mm diameter were cut with a biopsy punch (Kay Industries, Oyana, Japan). The discs were floated on 1.5 mL of distilled water in wells of a white 1.5-mL sample tube stand (Assist, Tokyo, Japan). A single adult female that had been starved for 2 to 3 h was placed on each leaf disc. The sample tube stand was covered with ABI Prism Optical Adhesive Cover (Applied Biosystems, Foster City, CA, USA), and a few tiny holes for air were made with a 27-G fine injection needle. Thrips were allowed to feed and oviposit for 4 days at 22°C. Eggs were stained with trypan blue as described previously [58].

### Counting of the thrips population

Three-week-old *Arabidopsis *plants or 10-day-old *B. rapa *plants grown in soil covered with fine zirconia beads (Nikkato Co., Osaka, Japan; 0.4 mm in diameter to make it easy to find the thrips) were placed in a cylindrical acryl chamber as above. Twenty adult females were put on each plant. After 2 weeks, the adults, larvae, and pupae were counted.

### Choice assay

Three-week-old WT and *coi1-1 *plants grown in soil covered with fine zirconia beads in a white pot (255 × 145 × 120 mm; Appleware, Osaka, Japan) were used for a choice assay in a cylindrical acryl chamber as above. Each pot held four plants (two of each type) separated by 150 mm. One hundred adult females were deposited halfway between the plants and allowed to move freely. After 2 days, the thrips on each plant were counted.

### Quantitative reverse transcription PCR

Twenty-five female adult thrips fed on five 2-week-old *B. rapa *plants at the rosette stage for 1, 2, or 5 days in a closed container with air vents. Experiments were repeated twice. After feeding, the plants were frozen in liquid nitrogen. Total RNA (2 μg) isolated with Trizol reagent (Invitrogen, Carlsbad, CA, USA) and an RNeasy MinElute Cleanup Kit (Qiagen, Valencia, CA, USA) was treated with RNase-free DNase (Takara) to eliminate genomic DNA. First-strand cDNA was synthesized with random oligo-hexamers and Superscript III reverse transcriptase according to the manufacturer's instructions (Invitrogen). Quantitative real-time PCR was carried out with Power SYBR Green PCR Master Mix (Applied Biosystems) using the first-strand cDNA as a template on a sequence detector (ABI Prism 7900HT, Applied Biosystems). Expression of *BrACT2 *was used for normalization. Nucleotide sequences of the gene-specific primers were as follows: *BrVSP2 *(forward, 5'-GACTCCAAAACGGTGTGCAAA-3'; reverse, 5'-AGGGTCTCGTCAAGGTCAAAGA-3'); *BrLOX2 *(5'-TCCCCACTTCCGCTACACC-3'; 5'-AATACTTTCCGGGCCAGAAAC-3'); *BrAOS *(5'-GATCTCCCCATCCGAACCAT-3'; 5'-AACTCCTCGGGTTTTTGCTTG-3'); *BrAOC2 *(5'-GCCGGTCTCTGTGTCTTGATC-3'; 5'-ACGGACAGGTGGCCATAGTC-3'); and *BrACT2 *(5'-ACCCAAAGGCCAACAGAGAG-3'; 5'-CTGGCGTAAAGGGAGAGAACA-3').

### Jasmonate quantification

JA and its methyl ester were quantified as described previously [47], except that an HP6890 gas chromatograph fitted to a quadrupole mass spectrometer (Hewlett-Packard, Wilmington, DE, USA) was used. Approximately 1 g of each *B. rapa *plant with or without thrips feeding was used for quantification. Three independent samples were analyzed.

### Measurement of the area of feeding scars

The area of thrips feeding scars on the surface of each *B. rapa *leaf was measured using WinROOF software, version 5.8.1 (Mitani Corporation, Tokyo, Japan), on digitized images taken under a VHX-200 digital microscope (Keyence, Osaka, Japan).

### Statistics

The results of thrips oviposition, population density and feeding activity were respectively subjected to Student's *t*-test or analysis of variance (one-way ANOVA) followed by Tukey-Kramer HSD test. The result from choice assay was subjected to a *χ*^2 ^test; the null hypothesis was that thrips exhibited a 50:50 distribution over WT and *coi1-1 *plants. These analyses were performed the JMP software, ver. 5.1 (SAS Institute, Inc., Cary, NC, USA).

### Accession numbers

The GenBank accession numbers for the genes mentioned in this article are as follows: *BrVSP2 *(EX101964), *BrLOX2 *(EX100417), *BrACT2 *(EX137335), *BrAOS *(EX104579), *BrAOC2 *(EX125486).

## Authors' contributions

HA planned the study and carried out the feeding assay, thrips performance and preference analyses, and wrote the manuscript. TS and SK performed thrips performance and preference analyses and participated in final writing of the manuscript. JO did the measurement of the area of feeding scars. MN and YN did the gene expression analyses of Brassica. SS performed JA quantification. ST and MK planned the study and participated in its coordination and final writing of the manuscript. All authors read and approved the final manuscript.
